# Effect of Tricalcium Aluminate on the Physicochemical Properties, Bioactivity, and Biocompatibility of Partially Stabilized Cements

**DOI:** 10.1371/journal.pone.0106754

**Published:** 2014-09-23

**Authors:** Kai-Chun Chang, Chia-Chieh Chang, Ying-Chieh Huang, Min-Hua Chen, Feng-Huei Lin, Chun-Pin Lin

**Affiliations:** 1 Graduate Institute of Clinical Dentistry, School of Dentistry and National Taiwan University Hospital, National Taiwan University, Taipei, Taiwan; 2 Institute of Biomedical Engineering, National Taiwan University, Taipei, Taiwan; University of Akron, United States of America

## Abstract

**Background/Purpose:**

Mineral Trioxide Aggregate (MTA) was widely used as a root-end filling material and for vital pulp therapy. A significant disadvantage to MTA is the prolonged setting time has limited the application in endodontic treatments. This study examined the physicochemical properties and biological performance of novel partially stabilized cements (PSCs) prepared to address some of the drawbacks of MTA, without causing any change in biological properties. PSC has a great potential as the vital pulp therapy material in dentistry.

**Methods:**

This study examined three experimental groups consisting of samples that were fabricated using sol-gel processes in C3S/C3A molar ratios of 9/1, 7/3, and 5/5 (denoted as PSC-91, PSC-73, and PSC-55, respectively). The comparison group consisted of MTA samples. The setting times, pH variation, compressive strength, morphology, and phase composition of hydration products and ex vivo bioactivity were evaluated. Moreover, biocompatibility was assessed by using lactate dehydrogenase to determine the cytotoxicity and a cell proliferation (WST-1) assay kit to determine cell viability. Mineralization was evaluated using Alizarin Red S staining.

**Results:**

Crystalline phases, which were determined using X-ray diffraction analysis, confirmed that the C3A contents of the material powder differed. The initial setting times of PSC-73 and PSC-55 ranged between 15 and 25 min; these values are significantly (*p*<0.05, ANOVA and post-hoc test) lower than those obtained for MTA (165 min) and PSC-91 (80.5 min). All of the PSCs exhibited ex vivo bioactivity when immersed in simulated body fluid. The biocompatibility results for all of the tested cements were as favorable as those of the negative control, except for PSC-55, which exhibited mild cytotoxicity.

**Conclusion:**

PSC-91 is a favorable material for vital pulp therapy because it exhibits optimal compressive strength, a short setting time, and high biocompatibility and bioactivity.

## Introduction

The pulpal tissue in teeth has various functions, including (1) physiological deposition of dentin by odontoblasts; (2) nutritional supply through microcirculation; (3) protection and sensation of nerve endings in dentin, and (4) repair under stimulation by forming tertiary dentin.[1] When pulpal tissue is subjected to trauma, caries, or iatrogenesis, the tooth can be treated with root canal treatment or pulp capping to prevent further pulpal inflammation or infection. Although the success rate of root canal treatment is high, the absence of pulpal tissue prevents teeth sensation or further repair.[2] To maintain the functions of pulpal tissue, vital pulp therapy (VPT) is a less invasive alternative treatment to root canal therapy. VPT involves direct pulp capping or partial pulpotomy, in which a biomaterial is used to maintain the vitality of the pulp of a tooth and establish an environment in which the dentin-pulp complex can form.[3] An ideal material for VPT must exhibit favorable sealing ability, nontoxicity to pulp tissue, antibacterial properties, high biocompatibility, stability in tissue fluids, a short setting time, adequate mechanical strength, and favorable handling properties.[4]

Numerous materials, such as zinc oxide eugenol, glass ionomer or resin-modifed glass ionomer, and calcium hydroxide, have been used in VPT[4]; however, none of these materials have achieved the requirements for an ideal material.[5]–[8] Calcium silicate cements, such as Portland cement or mineral trioxide aggregate (MTA), currently exhibit substantial potential for use as biomaterials in VPT.[9], [10] MTA is a bioactive material that features excellent apatite-forming ability; thus, it exhibits a substantial clinical advantage over traditional cements.[11] In addition, numerous studies have shown that MTA exhibits excellent sealing ability, a high pH, radiopacity, biocompatibility, and an ability to stimulate dentin matrix protein expression.[12]–[15] However, the poor handling properties and long setting time of MTA limit its clinical application.[4], [16] Effort is required to overcome the shortcomings of MTA as a biomaterial used in VPT.

In previous studies, we developed a partially stabilized cement (PSC) that exhibits similar properties to MTA.[17] PSC is an innovative biomaterial developed to reform some of the weaknesses of MTA. PSC is a refined calcium silicate cement of which the major chemical constituents are tricalcium silicate (3CaO•SiO_2_; C3S), dicalcium silicate (2CaO•SiO_2_; C2S), tricalcium aluminate (3CaO•Al_2_O_3_; C3A), and calcium aluminoferrite (4CaO•Al_2_O_3_•Fe_2_O_3_; C4AF), with specific ratios of each component. Among these components, C3S is associated with long-term mechanical strength. However, C3S and C2S exhibit long setting times and low mechanical strength at the early stages of hydrated calcium silicate cement.[18] Nevertheless, C3A exhibits the fastest hydration rate and provides the initial mechanical strength of calcium silicate cement.[19] Therefore, the C3A/C3S ratio may play a crucial role in the early hydration reaction of PSC, producing an accelerating effect on setting time. The sol-gel process is a useful method for preparing ceramics that enables easy control of the compositions of mixtures. Therefore, PSCs with different molar ratios of C3A were fabricated using a one-step sol-gel process featuring a low processing temperature, high chemical homogeneity, uniform phase distribution in a multicomponent system, and high reactivity of the product.[17], [20]

The purposes of this study were to investigate the effect of C3A on PSCs and compare the PSCs with MTA by using X-ray diffraction analysis (XRD) and scanning electron microscopy (SEM) to observe the microstructures and hydration behavior of PSCs in a physiological environment and by evaluating the pH variation, setting time, mechanical properties, and biocompatibility of the cements.

## Materials and Methods

### Material preparation

**Figure 1:**
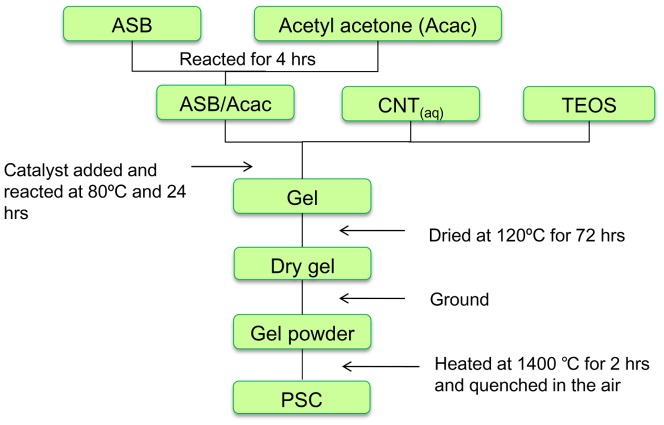
Schematic diagrams of the preparation of PSC. ASB  =  aluminum s*ec*-butoxide; CNT  =  calcium nitrate; TOES  =  tetraethyl orthosilicate; PSC  =  partial stabilized cement.

PSC powder was prepared using the sol-gel process as previously described.[17] Figure 1 shows schematic diagrams of the preparation. Aluminum *sec*-butoxide (ASB, Al(OBu^s^)_3_), an Al precursor mixed with acetylacetone (acac), was used as the complex ligand for modifying Al(OBu^s^)_3_. The mixture was stirred and reacted for 4 h in a complexing ratio (x) equal to 1. A Al(OBu^s^)_3_/(acac) complex was formed before conducting further sol-gel process reaction. The complexing ratio (x) represented the molar ratio of acac to Al(OBu^s^)_3_ (Al(OBu^s^)_3_/acac). After the surface of Al(OBu^s^)_3_ was modified, tetraethyl orthosilicate (Si(OEt)_4_) was added as a Si precursor to the solution, which was subjected to continual stirring. An aqueous solution of Ca(NO_3_)_2_ as a Ca precursor and Fe(NO_3_)_3_ as a Fe precursor was subsequently added to the solution. Ammonia water was added as a catalyst to facilitate reaction between alkoxides. A gel was formed and maintained for 24 h until gelation occurred. The gel was dried at 110°C, and then heated at 1400°C for 2 h and quenched in air. All of the reagents and chemicals used in this study were purchased from Sigma-Aldrich Co (St. Louis, MO, USA). White MTA was obtained from Dentsply, Tulsa Dental Products (Tulsa, OK, USA).

Three specimens (PSCs) were prepared in C3S (x)/C3A (y) molar ratios of 9/1, 7/3, and 5/5 (PSC-91, PSC-73, and PSC-55) by using sol-gel processes. De-ionized water (D.I. water) was then added to obtain PSC homogeneous pastes. The liquid-to-powder ratio (L/P) for all specimens was 0.5 mL/g. The mixtures were mixed for 5 min and then placed into a Teflon mold. The specimens were subsequently retrieved from the mold and stored in a sealed container with 100% relative humidity at 37°C to solidify further. The hydration products of all of the specimens were mixed with D.I. water and hydrated with a simulated body fluid (SBF)[21] solution at 4 h, 12 h, 1 day, 3 days, 7 days, 10 days, and 28 days. After incubating for a period of time, the specimens were soaked immediately in an anhydrous ethanol to stop the hydration reaction and enable them to be subjected to material tests.

### X-ray diffraction analysis

The crystalline phases of all specimens before and after hydration were determined using a Rigaku X-ray powder diffractometer (Rigaku Geigerflex, Japan) with CuK_α_ radiation (λ = 1.54 Å) and a Ni filter which was generated at 30 kV and 20 mA. The scanning rate of the specimens was 3°/min, and the scanning range (2θ) was 10° to 60°. The XRD patterns were collected and analyzed according to a model automatched to the standard JCPDS database by using Jade 6.0 software.

### Scanning electron microscope observation

The microstructures of the hydration products on the specimen surfaces were examined using a field-emission scanning electron microscope (FE-SEM, Hitachi S-4700) operated at 15 kV. Three specimens (PSCs) with different molar ratios of C3A were prepared for morphological observation conducted using a FE-SEM. After the specimens were air dried for 24 h at room temperature, the surfaces of the hydrated cement specimens were coated with a gold film by using sputtering physical vapor deposition and examined using the FE-SEM.

### Vicat setting time

The initial and final setting times of the PSCs and MTA were measured using a Vicat needle apparatus. This test was based on International Standard ISO 9597. The Vicat needle was cylindrical and 2.0 mm in diameter. The needle was initially fixed on a 100-g moveable rod and moved in a vertical alignment. Cement was placed in a mold and the needle penetrated 3–5 mm above the bottom of cement paste. The final setting time was determined using the needle (1.13 mm in diameter) loaded on a 300-g moveable rod, which no longer penetrated or indented the surface of the paste. Five cement specimens were measured, and the values are expressed as mean ± standard deviation (mean ± SD).

### pH variation

The pH values of all PSCs and the MTA were measured using the temperature-compensated electrode of a pH meter. Six samples of each cement were subjected to measurement. Each specimen was placed in a tube containing 10 mL of D.I. water and sealed in a container. The pH of the D.I. water in the tube was assessed using a pH meter at 2, 4, 12, 24, 72, 168, and 240 h. After each test, the samples were removed from the container and placed in a new container with the same volume of D.I. water.

### Mechanical strength measurements

The mechanical strength of all tested cements was evaluated according to the compressive strength. The specimen was placed into a cylindrical Teflon mold (4 mm in diameter × 6 mm in height) and stored in an environment of 100% relative humidity for 24 h at 37°C. Tensile strength data were collected by measuring the diameter and height of the specimens by using a micrometer. The specimens were fractured at a cross-head speed of 1.0 mm/min by using a universal testing machine (Instron 5566, Canton, MA, USA). The maximal load required to fracture each specimen was measured, and the compressive strength, σ, was calculated using the formula 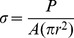
where *P* is the maximum load applied to the specimen in Newtons, and *A* is the area in millimeters squared. Statistical analysis was conducted using a one-way ANOVA in which *p* values (*) <0.05 indicated significance.

### Culture of human dental pulp cells

Primary human dental pulp cells were used in this study, and approved by the National Taiwan University Hospital (NTUH) Research Ethics Committee (REC) and all patients signed written informed consent, which was obtained from all subjects, dental pulp tissues were obtained from freshly extracted premolars and third molars without caries or pulpal diseases. A tissue explant technique was processed to cultivate dental pulp cells as described previously.[22], [23] Briefly, pulp tissues were minced into small pieces (approximately 1 mm^3^) and then digested with 3 mg/mL collagenase type I (Sigma, St Louis, MO, USA) and 4 mg/mL dispase (Sigma, St Louis, MO, USA) for 1 h at 37°C. The human dental pulp cells were cultured in Dulbecco's modified Eagle's medium (DMEM), which contained 4 mM L-glutamine, 4500 mg/L of glucose, 1 mM sodium pyruvate, and 1500 mg/L of sodium bicarbonate. The culture medium was supplemented with 10% fetal bovine serum, and the dental pulp cells were incubated in a humidified atmosphere of 5% CO_2_ at 37°C. When the dental pulp cells proliferated to 90% confluence in DMEM, the confluent cultures were detached using 0.25% Trypsin-EDTA and subcultured in a flask of DMEM to enable expansion. Cultured human dental pulp cells in passage number 3–10 were used for the following studies.

### Cytotoxicity assay and cell viability assay

The cytotoxicity of all tested cements were measured using the Cyto Tox Non-Radioactive Cytotoxicity Assay detection kit (Promega, Madison, WI, USA). The lactate dehydrogenase (LDH) activity was determined using a spectrophotometric assay. Methods for determining LDH involved combining tetrazolium salts with diaphorase. The chemical reactions of the assay are listed as follows:
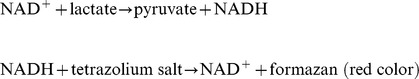



The cell cytotoxicity percentage was calculated by quantifying the amount of LDH in the medium from dead cells and dividing the result by the total amount of LDH in the medium and target cell lysate in the sample. Cell cytotoxicity results are expressed as the percentage of LDH released. The cytotoxicity of the cement was tested in accordance with ISO 10993-5. Briefly, 0.2 g of the sample was soaked in 1 mL of DMEM and incubated in 37°C for 72 h. Dental pulp cells were seeded at a density of 5×10^3^ cells per well in a 96-well culture plate for 24 h. After 24 h, the extract solution of the sample containing the medium at various concentrations was then added to the culture plate. The plates were incubated for 24 h and 72 h. The LDH released from the medium was measured using an ELISA reader (optical density at 490 nm).

Dental pulp cells were used in a cell proliferation and viability assay. Cells at 3×10^3^ cells/well were cultured in a 96-well culture plate containing 100 µL of DMEM per well for 1 day and 3 days. The cell viability of all tested cements was assessed by using a water-soluble tetrazolium salt-1 (WST-1) cell proliferation assay kit (Roche Diagnostics, Mannheim, Germany). This assay depends on cleavage of WST-1 by mitochondrial dehydrogenase in viable cells. The formazan dye produced by viable cells can be quantified. The number of viable cells was measured colorimetrically by using an ELISA reader at an absorbance (optical density, O.D.) of 440 nm. The results were expressed as the mean O.D. of experimental groups (n = 6) vs. negative control (normal DMEM). The mean O.D. of the control group was set to represent 100% cell viability.

### Alizarin red S staining

Mineralization of human dental pulp cells was assessed using Alizarin Red S staining (Sigma-Aldrich, St. Louis, MO, USA). Human dental pulp cells (5×10^3^ cells/well) were cultured in DMEM containing 10% FBS and treated with the extracts of all tested cements for 21 days for a mineralized nodule assay. The pulp cells were cultured with a culture medium containing extracts of the cements, and the culture medium was replaced every 3 days. After 21 days of treatment, the cells were rinsed twice with phosphate-buffered saline (PBS), fixed with 4% paraformaldehyde for 15 min at room temperature, and stained with a 2% (w/v) Alizarin Red S staining solution at a pH level of 4.2 and a temperature of 37°C. Images of Alizarin Red S staining were viewed and photographed under a light microscope.

### Statistical analysis

Data are expressed as the mean ± standard deviation (SD). Statistically significant differences from the control group were determined using a one-way factorial ANOVA. Differences with *p* values (*) <0.05 were considered significant.

## Results

### X-ray diffraction analysis

**Figure 2:**
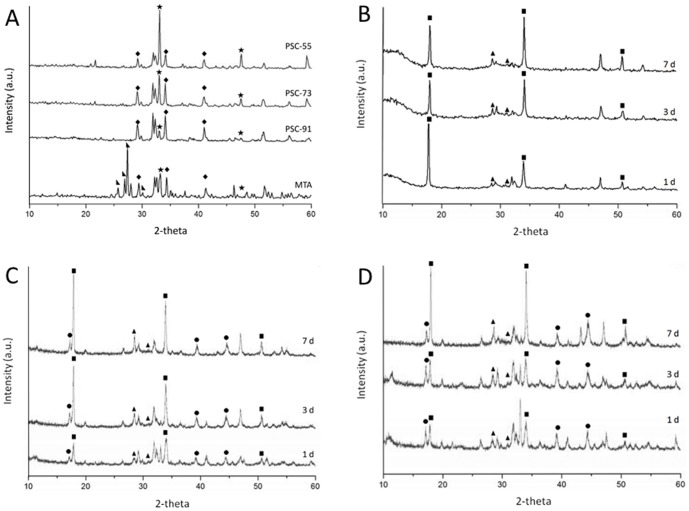
XRD powder patterns of unhydrated cements and hydrated PSCs stored in D.I. water for 1, 3 and 7 day. (A) the unhydrated of PSC-91, PSC-73, and PSC-55 after calcined at 1400°C for 2 h and unhydrated MTA; (B) the hydrated patterns of PSC-91; (C) the hydrated patterns of PSC-73; (D) the hydrated patterns of PSC-55. [★ C3A; 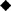 C3S; 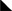 Bi_2_O_3_; ▪ Ca(OH)_2_; ▴ CSH; • C_3_AH_6_].

Figure 2 shows the XRD powder patterns of unhydrated and hydrated cements stored in D.I. water. Unhydrated PSC powder was obtained using sol-gel processes after calcination at 1400°C for 2 h and was characterized using XRD (Figure 2 A). Crystalline phases were characterized using standard data from the JCPDS database. Most of the diffraction peaks were identified as structures of C3S, C2S, C3A, and C4AF, which are the major components of PSC. The peaks at the position 2θ = 33.2 and 47.6° corresponded to a C3A structure, and the peak intensity increased as the C3A molar ratio increased. The peaks at 2θ = 23.2°, 32.1°–32.7° corresponded to C2S and C3S structures. A similar pattern was observed among the unhydrated MTA samples. In addition, the peak at 2θ = 37.3° and 53.9° corresponded to a CaO structure, and that at 2θ = 27.4° corresponded to a Bi_2_O_3_ structure.

For the hydrated PSCs, three phases of C3S, C2S, and C3A were identified at the same peaks at 2θ. However, the relative intensities of the peaks of these three phases decreased over time after the hydration reaction, as shown in Figure 2 (B)–(D). Portlandite hydrated products and calcium silicate hydrate (CSH) were observed in each experimental group after the samples were stored in D.I. water. Portlandite is a major hydration product of calcium silicate cement. The peaks at the position 2θ = 18° and 34.1° corresponded to Ca(OH)_2_, and the relative intensity of the peak increased as the degree of hydration increased. CSH (2θ = 28.6°, 29.1°, and 31.6°) was identified in all PSCs because of the hydration of C3S. Figure 2 (C) and Figure 2 (D) show the Ca_3_Al_2_(OH)_12_ (C_3_AH_6_) hydration product (2θ = 17.3°, 39.2°, and 44.4°). The relative intensity of the peak of PSC-55 was higher than that of PSC-73 because the amount of C3A in PSC-55 is greater; the intensity of the peak of PSC-91 was unclear (Figure 2 (B)).

### Characterization of hydration products soaking in simulated body fluid by using scanning electron microscopy

**Figure 3:**
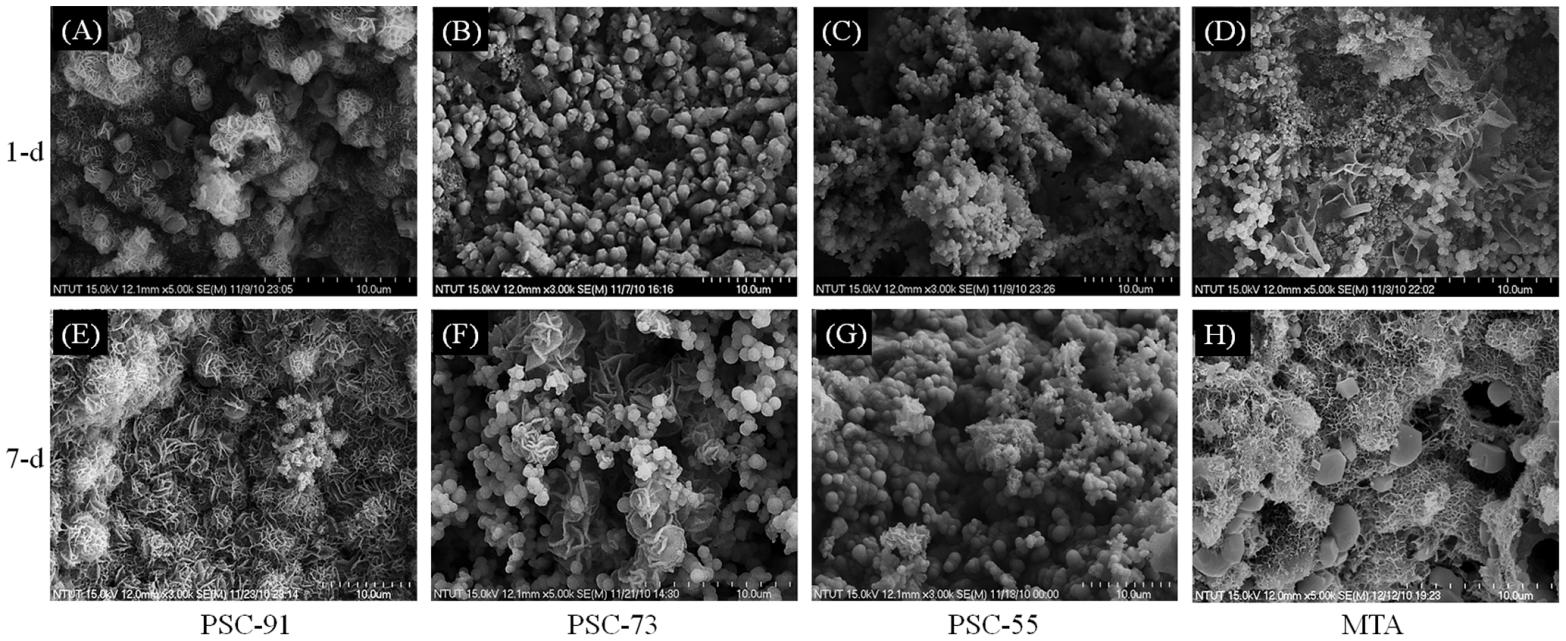
SEM micrograph of the surface of specimens stored in simulated body fluid (SBF) various durations. Soaked in SBF after 1 day: (A) PSC-91 (B) PSC-73 (C) PSC-55 (D) MTA; Soaked in SBF for 7 days: (E) PSC-91 (F) PSC-73 (G) PSC-55 (H) MTA

The PSC specimens, of which the microstructures are illustrated in Figure 3, were stored in an SBF environment for 1 day and 7 days. Morphological observations indicated that the hydrated PSC-91 soaked in the SBF for 1 day exhibited a cubic microstructure containing mesh-like crystals, as shown in Figure 3 (A). Hydroxyapatite-like crystals, which covered the specimen surface, were the hydration products of PSC-91; no cubic structure was observed. By contrast, the hydrated PSC-73 and PSC-55 that soaked in SBF for 1 day exhibited both a slight amorphous silk-like structure and a crystalline hexagonal structure, as shown in Figures 3 (B) and 3 (C). A silk-like structure is the initial hydration product of calcium silicate with a low Ca/Si ratio and crystallinity, whereas a crystalline hexagonal structure is a product of portlandite, which has a crystallinity that is higher than that of CSH. Figure 3 (E) shows the surface morphology of PSC-91 hydrated in SBF for 7 days; a large portion of the surface was covered with mesh-like HAp crystals. A hydroxyapatite-like structure began to form after only 1 day. After 7 days, aging completely covered the PSC-91 and MTA surface, as shown in Figure 3 (D) and (H). Figure 3 (G) shows that mesh-like crystals embedded the ball-like structure in the matrix of the PSC-55 specimen that was soaked in SBF for 7 days. The hydration products of PSC-55 included more hydroxyapatite-like crystals compared with the specimen that was soaked in SBF for 1 day. A gradual change in the microstructure of PSC-73 was observed using FE-SEM, as shown in Figure 3 (F); additional ball-like crystals and few hydroxyapatite-like crystals covered the matrix.

### Physicochemical properties of the cements

**Figure 4:**
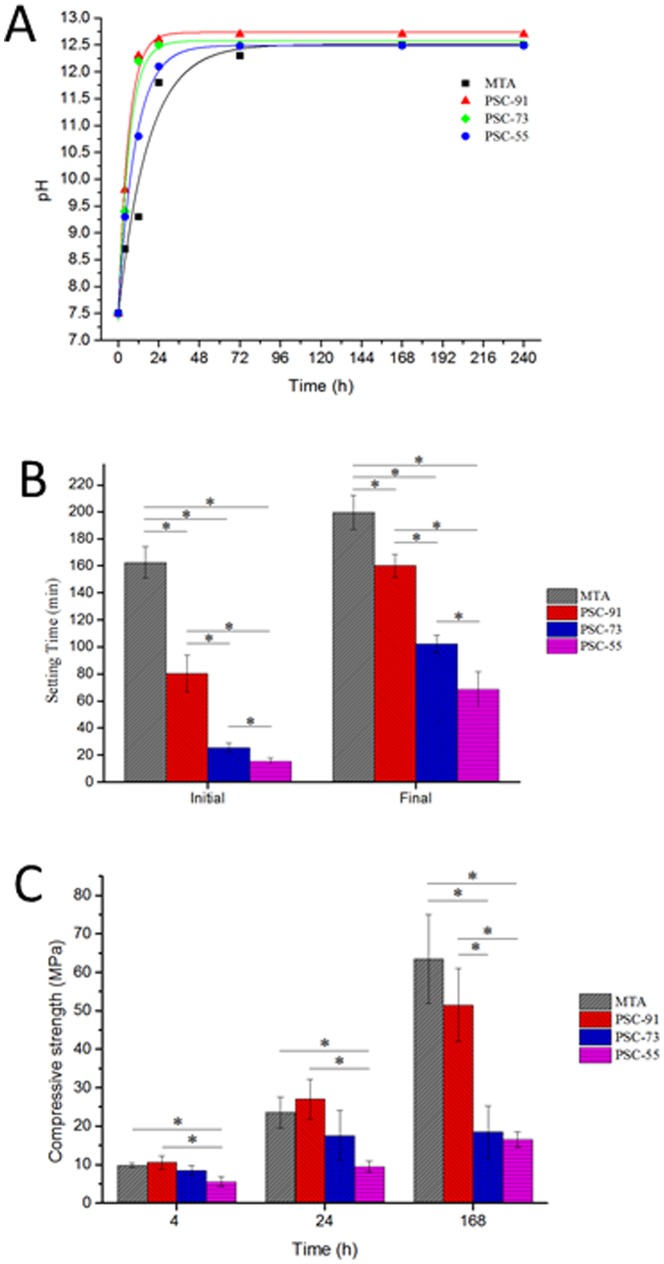
The pH value and physical properties of all tested cements. (A) pH values variation of all tested cements at various time intervals. (B) The initial and final setting times of PSC-91, PSC-73, PSC-55 and MTA. (C) Compressive strength of PSC with different C3S and CSA content and MTA at 4-h, 24-h and 168-h.

The physicochemical properties of all tested cements are shown in Figure 4. The change in pH as a function of time for all materials is shown in Figure 4 (A). The initial pH value of all tested cements after mixing was approximately 7.5 and rose to 12.4–12.7. The initial pH value of PSC-91, PSC-73, and PSC-55 at 4 h was higher than that of the MTA, and the alkalinity of these PSCs tended to increase over time. All cements exhibited a high alkaline pH. The highest value was that of PSC-91, and the mean was 12.7 at 240 h. The pH values of PSC-91, PSC-73, and PSC-55 did not differ significantly from those of MTA after 72 h.

The initial and final setting times of all of the materials are shown in Figure 4 (B). The initial setting times for PSC-91, PSC-73, and PSC-55 were between 15.5 and 80.5 min, and the final setting times ranged between 68.5 and 160 min. C3A exhibited an accelerated setting effect during hydration of the PSCs compared with MTA. When the molar ratios of C3A in the PSC were increased from 10% to 50%, the initial and final setting times of the PSCs decreased dramatically compared with those of MTA.

Figure 4 (C) shows the compressive strength of all of the tested cements. PSC-91 and PSC-73 exhibited similar compressive strength in the early stage in 4 h. The compressive strength of PSC-55 was slightly lower than that of the other experimental samples at 4 h. After being set in D.I. water for 24 h, PSC-91 exhibited higher compressive strength (27.1 MPa) than PSC-73 and PSC-55 did. PSC-91 exhibited the highest compressive strength among the PSCs, 51.5 MPa, after it was set for 168 h. Reducing the amount of C3A increased the mechanical strength of the PSCs. The compressive strength of PSC-73 and PSC-55 did not differ significantly after the specimens were set for 168 h. The results revealed that the content of C3A in the PSCs exerted an obvious effect on the compressive strength of the cement.

### Biocompatibility assay

**Figure 5:**
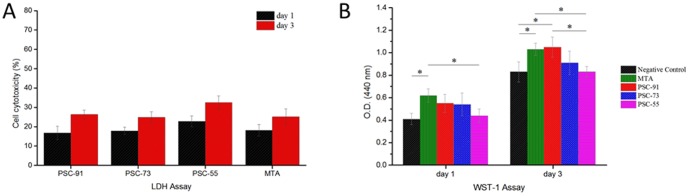
(A) Cytotoxicity assessment of PSC-91, PSC-73, PSC-55 and mineral trioxide aggregate (MTA) by LDH assay according to ISO-10993 protocol standard. All tested cements on dental pulp cells were evaluated by LDH assay on 1day and 3 days. Each bar illustrated average absorbance (A490 nm) ± SD. No significant differences between PSC-91, PSC-73 and MTA (P>0.05); (B) Cell viability evaluation by WST-1 assay. Each bar illustrated average absorbance (A440 nm) ± SD.

The cytotoxicity of all tested cements was determined by conducting an LDH assay. The cytotoxicity of the control group and all of the tested biomaterials increased over time, as shown in Figure 5 (A). PSC-91 and PSC-73 exhibited no statistically significant difference from the MTA at 1 day and 3 days. However, the cytotoxicity of PSC-55 was significantly higher than that of the other experimental samples at 1 day and 3 days. The results of the LDH assay indicated that the PSCs exhibited low levels of cell cytotoxicity.

Cell viability was evaluated according to the mitochondrial function (WST-1 assay) of the cells. The O.D. value was directly proportional to cell number. As shown in Figure 5 (B), the O.D. value of MTA group was significant higher than that of the negative control group at day 1 and 3. The O.D. value of PSC-91 was significantly increased compared with negative control group at day 3. The cell numbers of PSC-55 at 1 day and 3 days exhibited a statistically significant difference (p<0.05) from those of MTA. Increasing the molar ratios of C3A in the PSCs decrease the activity of mitochondria (cell viability).

### Mineralized nodule formation

**Figure 6:**
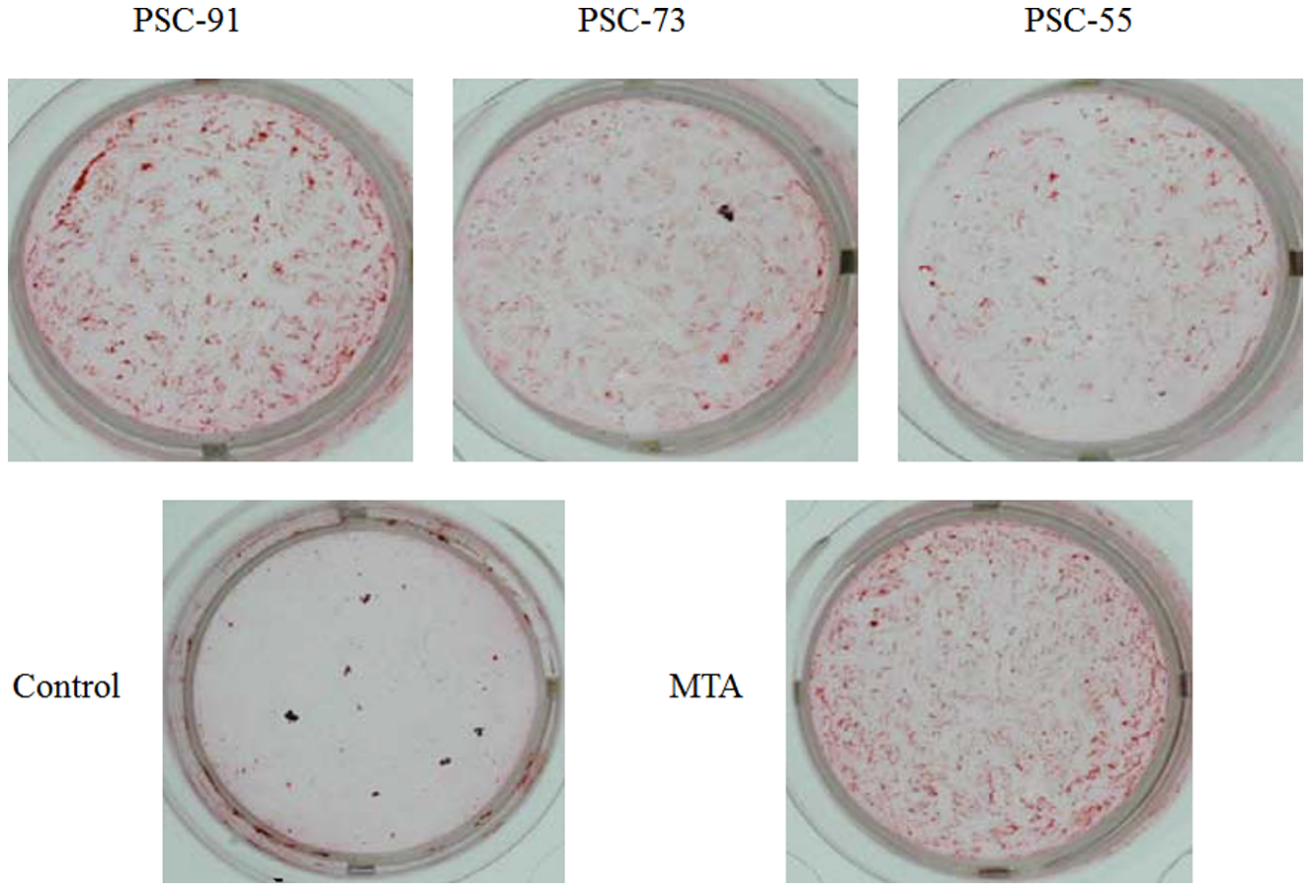
The evaluation of biomineralization in human dental pulp cells by Alizarin Red S staining.

The effect of all of the tested cements on the formation of calcification nodules in human dental pulp cells that were incubated for 21 days and examined using Alizarin Red S staining is shown in Figure 6. PSC-91 and MTA exhibited a significant increase in the area of calcified nodules compared with PSC-73 and PSC-55, whereas no clear mineralization was observed in the control cells.

## Discussion

In this study, three PSCs containing C3A in different ratios were fabricated using sol-gel processes. The sol-gel process is a useful method for preparing ceramics and glass [24] and features high chemical homogeneity, uniform phase distribution in multicomponent systems, high reactivity of the product, and low synthesis temperatures. According to the XRD results (Figure 2 (A)), the major components of the synthesized unhydrated PSCs were C3S, C2S, and C3A; this composition is similar to those of MTA and portlandite. The XRD peak intensity of C3A increased as the molar ratio of C3A in PSC increased, indicating that a proportion of raw materials reacted homogenously during the sol-gel process.

C3S and C2S are two major components of PSC. Their hydration reaction can be expressed using Formulas (1)–(3). The hydration products are CSH and calcium hydroxide (CH). The C3A is substantially influenced by the early hydration behavior of PSC. The hydration of C3A is rapid and its hydration reactions can be expressed using Formula (4).[25] The hydration process in this reaction involves several hydrates; (Ca_3_Al_2_(OH)_12_; C_3_AH_6_) is one of the hydrates and is the most stable at high temperatures.

The hydration reaction of each component of PSC is expressed as follows:
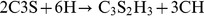
(1)

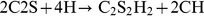
(2)

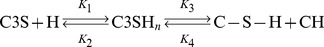
(3)

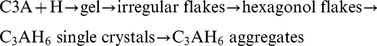
(4)where [A  =  Al_2_O_3_; C  =  CaO; CH  =  Ca(OH)_2_; H  =  H_2_O; S  =  SiO_2_; C-S-H is amorphous hydrogen having variable composition in terms of Ca/Si ratio and H_2_O/SiO_2_ ratios].

The hydrolysis of calcium silicates produces calcium hydroxide and creates a less basic calcium silicate hydrate.[26] Calcium hydroxide precipitated during the hydration of PSC. The presence of calcium hydroxide causes the hydrated PSC to be highly alkaline (pH 12.5). All of the cements evaluated in this study increased the pH values (7.5 at 12.7) through the release of hydroxyl ions. The more C3A in PSC, the slower pH value rising in the immersed solution. The curve of pH versus immersion time; where slope of the initial stage was different depends on the C3A molar ratio in PSCs. Figure 4 (A) shows the rising curve: PSC-91>PSC-73>PSC-55>MTA. MTA contains gypsum, which hindered the hydration reaction, causing the pH value to rise at slowest rate. The highly alkaline environment has an antibacterial effect and promotes cell remineralization.[27]

One of the most clinically relevant factors is the setting time of biomaterials, which is affected by numerous factors, such as the L/P ratio, particle size of the cement, chemical content of the cement, and cement additives. A long setting time may prevent the material from being held at the operation site, causing a lack of mechanical strength required for initial support.[28] The initial setting time of MTA is approximately 165 min. Because of this long initial setting time, a two-step procedure must be used when applying MTA in VPT. C3A is the most reactive component of Portland cement, and increasing the molar ratio C3A in PSCs may reduce the setting time and enhance the initial compressive strength. PSC-55 has short initial (15.5 min) and final (68.5 min) setting times (Figure 4 (B)) because the ratio of C3A to C3S is high; however, the compressive strength of PSC-55 and PSC-73 was lower than that of the other experimental groups during the 7-day hydration reaction, as shown in Figure 4 (C). By contrast, C3S and C2S play a crucial role in cement mechanical strength, and C2S hydration occurs more slowly than C3S hydration does, contributing to the long-term strength of the cement.

Calcium silicate materials exhibit dissolution and precipitation behaviors during the hydration reaction. The calcium and hydroxyl ions released from materials reacts with phosphate to form a hydroxyapatite structure.[29] Hydroxyapatite plays a crucial role in tissue regeneration and maintaining function because of its bioactive surface.

According to the SEM observation, the hydroxyapatite layer precipitated on the surface of all of the PSCs after they were exposed to the SBF environment for 7 days, as shown in Figure 3. The mesh-like apatite layer was observed in PSC-91 and MTA at 1 day and 7 days. However, this layer was not observed in PSC-73 and PSC-55 until the samples were immersed in SBF for 7 days. In addition, the hydration products were examined using XRD, and the patterns indicated that Ca-P hydrated products and calcium carbonate (CaCO_3_) were present in each experimental group after the samples were soaked in SBF. In addition, the characteristic peaks of hydroxyapatite crystals were present in the PSC-91 and MTA groups, suggesting that hydroxyapatite precipitated on the surfaces of all of the specimens after they were soaked in SBF for 7 days. Both the SEM and XRD results indicated that the C3A and C3S in PSCs facilitated the precipitation of a hydroxyapatite-like active layer, suggesting that the PSCs feature favorable in vitro bioactivity. The time required for C3A and C3S to induce a hydroxyapatite-like structure increases when the C3A molar ratio is over 30% in PSC. Small round particles were observed on the surface of the PSCs after they were immersed in SBF for various durations. XRD analysis revealed that the hydration products were calcium-deficient carbonated apatite or had a calcium phosphate structure.

The biocompatibility of dental materials can be evaluated using numerous mammalian cell culture methods. Extracts of all of the tested cements were used in this study. According to the ISO 10993-5 standard, all of the tested cements were evaluated using in vitro tests of cytotoxicity, and the response of cells to the extracts were assessed. Numerous studies have reported that MTA features high biocompatibility.[30]–[32] In this study, MTA was used as a comparative biomaterial because PSC is chemically similar to Portland cement, which exhibits a biological response similar to that of MTA.[33] Figure 6 (A) indicates that PSC-55 contained a substantial amount of C3A, exhibiting a statistically significant difference in the LDH assay compared with the other groups at 1 day and 3 days of incubation. Liu et al. determined that adding 0%–15% C3A into C3A and C3S mixtures yields no significant cytotoxicity within the extract concentration range between 3.125 mg/mL and 100 mg/mL.[34] The extract concentration in this study was 100 mg/mL and, according to the results of the cell viability assay, all of the PSCs exhibited no cytotoxicity and did not influence cell function for a long period of time. Finally, LDH and WST-1 assay kits were used in this study, and the results indicated that all of the PSCs and MTA are biocompatible. Mineralized nodules were characterized by using Alizarin Red S staining. After 21 days of induction, we observed that mineralized nodules of human dental pulp cells formed. All of the tested cements induced mineralization of the pulp cells. In addition, the stain of mineralized nodules of PSC-91 and MTA was more intense under light microscopy. These results indicated that PSC-91 can facilitate the formation of mineralized nodules of human dental pulp cells.

In summary, increasing the C3A content from 30% to 50% significantly improved the setting time of the PSCs; however, the mechanical strength and biological properties of the PSCs deteriorated. According to the results of this study, PSC-91 exhibits an appropriate setting time and high mechanical strength as well as a cellular response similar to that of MTA, a commercial product applied in VPT. Based on its physicochemical properties, in vitro biocompatibility, and bioactivity, PSC-91 can be applied in VPT.

## Conclusions

In this study, three PSCs containing C3A in different molar ratios were fabricated using sol-gel processes. Their physicochemical properties, bioactivity, and biocompatibility were characterized using XRD, SEM, pH-metry, an Instron machine, a Vicat needle apparatus, Alizarin Red S staining, and a cytotoxicity assay kit. The PSC containing 10% C3A (PSC-91) exhibited optimal compressive strength and a setting time shorter than that of MTA. The hydration properties of C3A in PSCs played a critical role at the early stages, and the results indicated that PSC-91 facilitated apatite formation when the cement was soaked in SBF. The results of the Alizarin Red S staining and biocompatibility assay indicated that PSC-91 exerts no effect on cell viability or cytotoxicity and facilitates the formation of mineralized nodules of human dental pulp cells. The physicochemical properties and biocompatibility of PSC-91 indicated that this material has considerable potential for use as a biomaterial in VPT. However, additional studies are required to explore the physical properties, antimicrobial properties, and in vivo effectiveness of this material.
